# Highly improved supercapacitance properties of MnFe_2_O_4_ nanoparticles by MoS_2_ nanosheets

**DOI:** 10.1038/s41598-021-87823-6

**Published:** 2021-04-16

**Authors:** Samira Sharifi, Kourosh Rahimi, Ahmad Yazdani

**Affiliations:** Condensed Matter Physics Group, Department of Basic Sciences, Tarbiat Modares University, Jalal-Ale-Ahmad Avenue, Tehran, Iran

**Keywords:** Surfaces, interfaces and thin films, Nanoscale materials, Supercapacitors, Energy

## Abstract

Manganese ferrite (MnFe_2_O_4_) nanoparticles were synthesized via a hydrothermal method and combined with exfoliated MoS_2_ nanosheets, and the nanocomposite was studied as a supercapacitor. X-ray diffractometry and Raman spectroscopy confirmed the crystalline structures and structural characteristics of the nanocomposite. Transmission electron microscopy images showed the uniform size distribution of MnFe_2_O_4_ nanoparticles (~ 13 nm) on few-layer MoS_2_ nanosheets. UV–visible absorption photospectrometry indicated a decrease in the bandgap of MnFe_2_O_4_ by MoS_2_, resulting in a higher conductivity that is suitable for capacitance. Electrochemical tests showed that the incorporation of MoS_2_ nanosheets largely increased the specific capacitance of MnFe_2_O_4_ from 600 to 2093 F/g (with the corresponding energy density and power density of 46.51 Wh/kg and 213.64 W/kg, respectively) at 1 A/g, and led to better charge–discharge cycling stability. We also demonstrated a real-world application of the MnFe_2_O_4_/MoS_2_ nanocomposite in a two-cell asymmetric supercapacitor setup. A density functional theory study was also performed on the MnFe_2_O_4_/MoS_2_ interface to analyze how a MoS_2_ monolayer can enhance the electronic properties of MnFe_2_O_4_ towards a higher specific capacitance.

## Introduction

There have been increasing demands in the past few decades for superior energy storage and conversion devices to address the basic energy-related needs of the ever-growing population in the world^1^. Therefore, it is indispensable to develop energy-storage devices with high energy capacities, long lifetimes, and high cycling stability to overcome the impending exhaustion of fossil fuel reserves and alleviate environmental concerns^2^. Supercapacitors are among the most-promising energy-storage devices owing to their longer lifespan than secondary batteries and their higher capacitance and reliability than conventional dielectric capacitors^2^. There are two classifications for supercapacitors based on their energy storage mechanisms: (1) electrochemical double-layer capacitors that accumulate charges at their electrode/electrolyte interface and (2) pseudocapacitors that handle charges via fast and reversible redox reactions on electrochemically active sites^3^. However, it is yet challenging to design and develop electrode materials to realize these anticipated features and efficiently store/deliver energy^4–6^.

There has been recently growing attention to two-dimensional (2D) layered materials for a variety of applications including energy production and storage, sensors, photocatalysts, etc.^7–9^. Recent developments suggest that 2D transition metal dichalcogenides (TMDs) such as MoS_2_, MoSe_2_, WS_2_, TiS_2_, NbS_2_, and VS_2_ have great potential to fill the gap between the current performance and the modern requirements of energy-storage devices as electrodes of electrochemical supercapacitors^10–13^. In general, TMDs make use of fast and reversible faradaic redox reactions (also known as pseudocapacitance) that involve ions and electrons in their charge storage mechanism^14,15^. In particular, few-layer MoS_2_ nanosheets have been found promising because of their large surface area, which acts as a substrate to hold other nanoparticles, and high thermal stability^16^.

Among various transition metals^17^, nickel, manganese, and cobalt are promising in the field of supercapacitors due to their high electrochemical activity and low cost as well as the abundance of their oxide/hydroxide compounds^18–24^. It has also been demonstrated that the spinel ferrites of these metals (MFe_2_O_4_, M is a transition metal) deliver much better electrochemical performance due to their richer valence electron, different redox states, synergistic effects between their metal ions, electrochemical stability, and chemical and mechanical stability, suitable for batteries and supercapacitors^14,25–29^. Recently, we have compared the supercapacitance of MnFe_2_O_4_, CoFe_2_O_4_, and NiFe_2_O_4_ nanoparticles, and found that MnFe_2_O_4_ exhibits better supercapacitance properties^14,15^.

It is thus interesting to make composites of MnFe_2_O_4_ nanoparticles and few-layer MoS_2_ nanosheets, as a 2D TMD, to utilize their synergistic effects to achieve improved electronic properties^30,31^. In such a composite, the MnFe_2_O_4_ nanoparticles would prevent the MoS_2_ nanosheets from restacking, and in a similar manner, the MoS_2_ nanosheets would act as a substrate on which the MnFe_2_O_4_ nanoparticles can be uniformly distributed so that they would not be agglomerated. The mutual effect would result ultimately in a larger active surface area that can promote electrolyte access and provide more channels for migration of ions and electrons^32^.

To the best of our knowledge, there is not yet any report on supercapacitance properties of the composite of MoS_2_ nanosheets (as a TMD) decorated with MnFe_2_O_4_ (as a metal ferrite) nanoparticles, and it is thus interesting to see how the probable synergistic effect of them can be useful to achieve improved electrochemical energy storage performance. Here, we reported the successful fabrication of the MnFe_2_O_4_/MoS_2_ nanocomposite on nickel foam via a facile hydrothermal method and tested the nanocomposite as a supercapacitor electrode.

## Experimental

Manganese(II) nitrate tetrahydrate (Mn(NO_3_)_2_.4H_2_O), iron(III) nitrate nonahydrate (Fe(NO_3_)_3_.9H_2_O), cetyltrimethylammonium bromide (CTAB), N-Methyl-2-pyrrolidone (NMP), acetonitrile, polyvinylidene difluoride (PVDF), activated carbon (AC), and hydrogen peroxide (H_2_O_2_) were purchased from Merck Co. (> 98%) and bulk molybdenum disulfide (MoS_2_, 99%) powder was purchased from Sigma-Aldrich Co, and the precursors were used without any further purification.

### Synthesis

**Figure 1:**
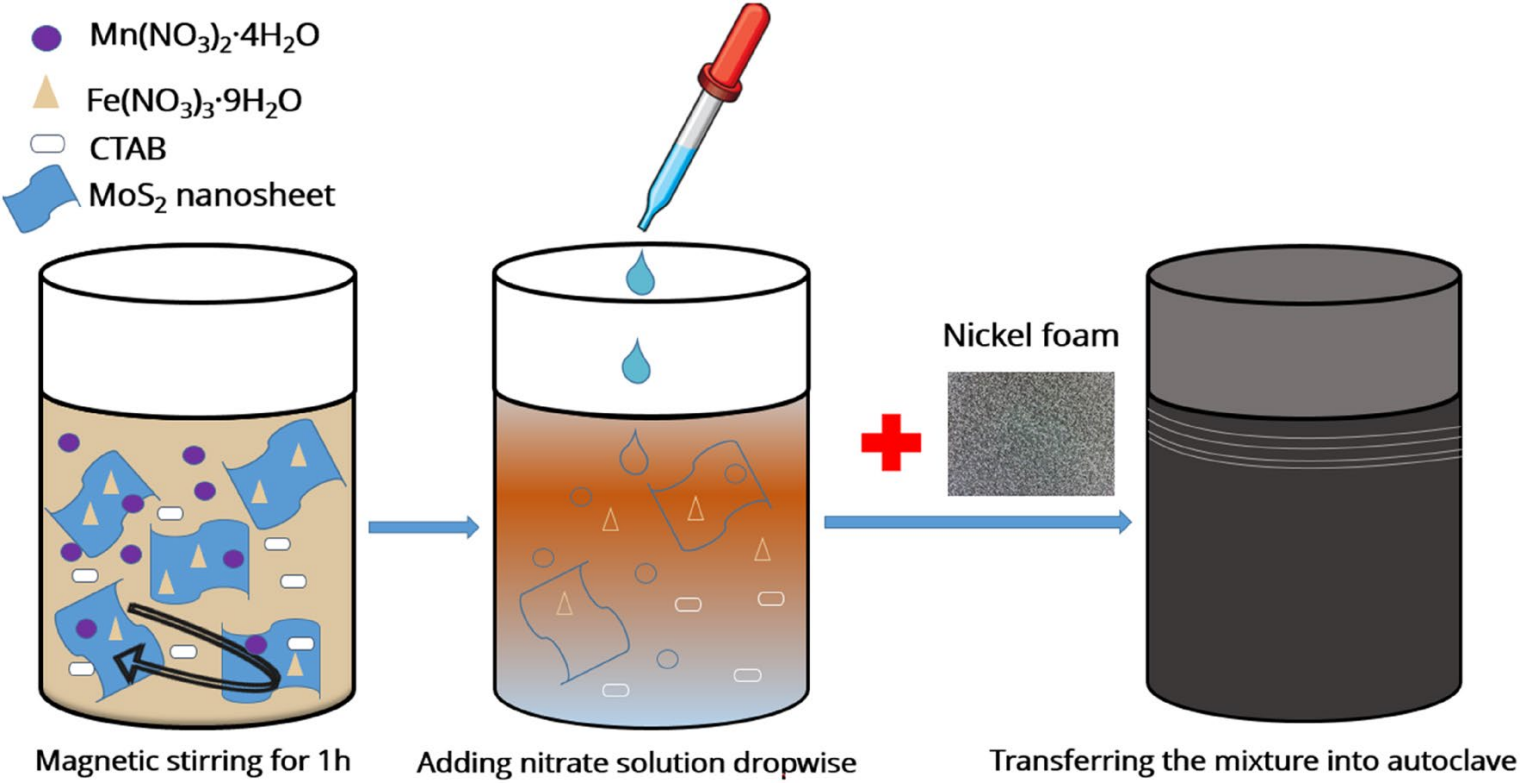
The procedure to synthesize the MnFe_2_O_4_/MoS_2_-coated Ni foam (drawn using Microsoft PowerPoint 2016, downloadable from www.microsoft.com).

Few-layer MoS_2_ nanosheets were exfoliated from bulk MoS_2_ powder in mixed solvents based on the work of Lu et al.^33^. First, 20 mg MoS_2_ powder was mixed in 0.5 ml acetonitrile and it was ground for 1 h. The obtained powder was mixed in a solution of 30 wt% H_2_O_2_ and NMP (H_2_O_2_:NMP volume ratio = 1:19), and it was stirred for 10 h at 35 °C to be exfoliated. The mixture was dried in a furnace for 5 h at 300 °C to evaporate NMP. To in-situ synthesize the MnFe_2_O_4_/MoS_2_ nanocomposite on a Ni foam substrate using a hydrothermal method, 0.1 g of the obtained MoS_2_ nanosheets was first dispersed into 40 ml deionized water, and 0.4 g Fe(NO_3_)_3_.9H_2_O, 0.125 g Mn(NO_3_)_2_.4H_2_O, and 0.125 g CTAB were dissolved into the dispersion by stirring for 2 h. Next, 1 ml of 25% ammonia solution was added into the prepared mixture under vigorous stirring until its pH reached ~ 9. The obtained mixture was transferred into a Teflon-lined autoclave and a nickel foam substrate, cut in the size of 1 × 2 cm^2^, and cleaned with deionized water, acetone, and ethanol, was put into it. The autoclave was subsequently heated in an oven at 180 °C for 15 h, and then it was allowed to cool to room temperature in ambient air. Finally, the MnFe_2_O_4_/MoS_2_-coated nickel foam was washed several times with deionized water and ethanol and dried in an oven at 80 °C for 2 h. For comparative purposes, MnFe_2_O_4_-coated nickel foam was also synthesized via a similar method without adding MoS_2_. The prepared substrates were used for further characterizations. Figure 1 shows a schematic of various steps followed in our synthesis procedure.

### Characterization

Crystalline structures of the samples were identified using a PANalytical X'pert MPD (Philips) diffractometer with a Cu-Kα radiation source (λ = 0.15406 nm). Structural fingerprints of the ferrites and the MoS_2_ nanosheets were investigated by a Takram P50C0R10 Raman spectrometer (Teksan Co., Iran) employing an Nd:YAG laser (λ_ex_ = 532 nm) at room temperature. To observe structural shapes ph of the prepared nanostructure, field-emission scanning electron microscopy (FESEM) and transmission electron microscopy (TEM) images were taken by MIRA3TESCAN-XMU and PHILIPS CM30 NETHERLANDS instruments, respectively. The elemental compositions of the samples were analyzed by energy-dispersive X-ray spectroscopy (EDS) mapping using a BRUKER XFlash 6 I10 instrument. The topographical information of MoS_2_ nanosheets was acquired by atomic force microscopy (AFM, Veeco Autoprobe CP-research). The optical properties of the nanocomposites were examined using a Unico 4802 UV–Vis photospectrometer.

### Electrochemical tests

The supercapacitive performance of the samples was investigated using a three-electrode setup containing the coated Ni foam substrate (1 cm^2^) as the working electrode, a square-shaped platinum sheet (1 cm^2^, 99.99%) as the counter electrode, and Ag/AgCl as the reference electrode in a 3 M KOH solution at room temperature. Although Ni foam shows a battery-like behavior^34^, we chose it because of its large specific surface area that can accommodate more parts of active materials. In this regard, the calculated specific capacitance is better not to be compared with other literature. Nevertheless, we aim at comparing the specific capacitances of our samples with each other to find how the incorporation of MoS_2_ nanosheets can enhance the specific capacitance of MnFe_2_O_4_ nanoparticles. The electrochemical measurements involved cyclic voltammetry (CV), galvanostatic charging/discharging (GCD), and electrochemical impedance spectroscopy (EIS) techniques using a VSP-300 Multichannel Potentiostat/Galvanostat/EIS instrument (Bio-Logic Science Instruments). The CV measurements were recorded at different scan rates (5–100 mV/s) within the potential window of 0–0.55 V. The GCD measurements were recorded at different current densities with the potential window of 0–0.4 V.

### Asymmetric two-electrode supercapacitor setup

An asymmetric two-electrode supercapacitor device was assembled by using activated carbon (AC) as the negative electrode and the MnFe_2_O_4_/MoS_2_ nanocomposite as the positive electrode. The electrodes were separated by a filter paper wetted with 3 M KOH solution as the electrolyte. The AC electrode was prepared from the activated carbon and PVDF, as a binder, with the weight ratio of 95:5 dispersed in NMP. The prepared dispersion was coated on a nickel foam substrate by a brush and the obtained electrode was dried in an oven at 60 °C for 10 h. The masses of the positive and negative electrodes were balanced according to the following equation^14^:

1 $$\frac{{m}_{+}}{{m}_{-}}=\frac{{C}_{S}^{-}{\Delta V}^{-}}{{C}_{s}^{+}{\Delta V}^{+}}$$

where m is the mass, C_s_ is the specific capacitance, ∆V is the potential window, and ( +) and ( −) denote the positive and the negative electrodes, respectively. The coated mass on the negative electrode was ~ 3 mg. The CV measurements were recorded at different scan rates (5–100 mV/s) within the potential window of 0–1.5 V. The GCD measurements were recorded at different current densities with the potential window of 0–1.5 V.

### Computational methods

First-principles calculations were performed in the framework of density functional theory (DFT), as implemented in the Quantum Espresso package (version 6.3)^35^, using the plane-wave basis set and ultrasoft pseudopotentials^36^. The spin polarization was included in both geometry optimizations and electronic structure calculations. The generalized gradient approximation (GGA) developed by Perdew, Burke, and Ernzerhof (PBE)^37^ was applied for electron exchange–correlation functionals with the on-site Coulomb repulsion U terms^38^ of U(Mn) = 3.9 eV and U(Fe) = 5.3 eV to reproduce experimental data^14^. The kinetic energy cutoffs for wavefunctions and charge densities were set to 50 and 450 Ry, respectively. To sample the first Brillouin zone for electronic structure calculations, we adopted the k-point grid of 9 × 9 × 1 for the unit cell of the MoS_2_ monolayer, 6 × 6 × 5 for the bulk MnFe_2_O_4_, and the k-point grid of 6 × 6 × 1 for the MnFe_2_O_4_/MoS_2_ interface. All structures were fully relaxed until the convergence criteria of energy and force became less than 10^–6^ Ry and 10^–3^ Ry/Bohr, respectively. All crystal images were produced by VESTA (version 3.4.5)^39^.

## Results and discussions

**Figure 2:**
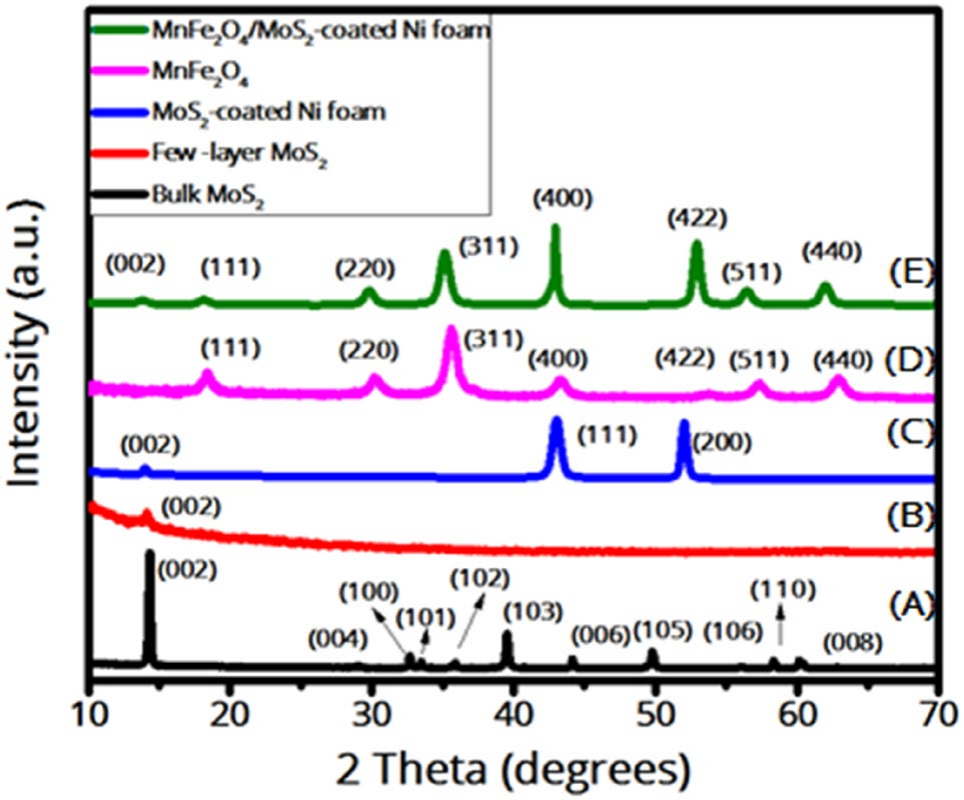
XRD patterns of (**A**) bulk MoS_2_, (**B**) exfoliated MoS_2_ nanosheets, (**C**) MoS_2_-coated Ni foam, (**D**) MnFe_2_O_4_ nanoparticles, and (**E**) MnFe_2_O_4_/MoS_2_-coated Ni foam.

The XRD patterns of the samples are shown in Fig. 2. The XRD pattern of the bulk MoS_2_ shows all the characteristic peaks corresponding to the hexagonal phase of MoS_2_ with the JCPDS card No. 00-037-1492. However, the XRD pattern of the exfoliated MoS_2_ nanosheets shows only the weak (002) diffraction peak, indicating the successful exfoliation of MoS_2_ into few-layer nanosheets. In the pattern of MnFe_2_O_4_ nanoparticles, the peaks at 2θ = 18°, 30°, 35°, 43°, 53°, 57°, and 63° correspond to (111), (220), (311), (400), (422), (511), and (440), which are attributed to the cubic spinel structure of MnFe_2_O_4_ with the space group of $Fd\stackrel{-}{3}m$ with the JCPDS card No. 96-591-0064^32^. No other peak is seen, indicating the purity of the prepared nanoparticles. To calculate the crystallite size and the lattice strain for the MnFe_2_O_4_ nanoparticles, the Williamson-Hall analysis formula was applied^40^

2 $${\beta }_{hkl }cos {\theta }_{hkl} =\frac{k\lambda }{D}+4\varepsilon sin {\theta }_{hkl}$$

where θ_hkl_ is the diffraction peak angle, ${\beta }_{hkl}$ is the full-width at half maximum of the (hkl) diffraction peak, D is the crystallite size, K is the shape factor approximated to 0.9, λ is the incident X-ray wavelength (1.5406 Å), and ε is the lattice strain^41^. Accordingly, D and ε are calculated from the Y-intercept and the slope of the line fitted on the plot of 4sinθ versus βCosθ, respectively^32,41^. Therefore, the crystallite size of the MnFe_2_O_4_ nanoparticles was calculated ~ 9.5 nm with a compressive strain of − 0.0144. In the XRD pattern of the MnFe_2_O_4_/MoS_2_-coated Ni foam, all of the peaks related to MnFe_2_O_4_ are seen with a peak at 2θ = 14° related to few-layer MoS_2_ nanosheets and two other peaks at 2θ = 43° and 53° corresponding to the Ni foam substrate. This indicates that the nanocomposite has been successfully prepared, with no other impurities.

**Figure 3:**
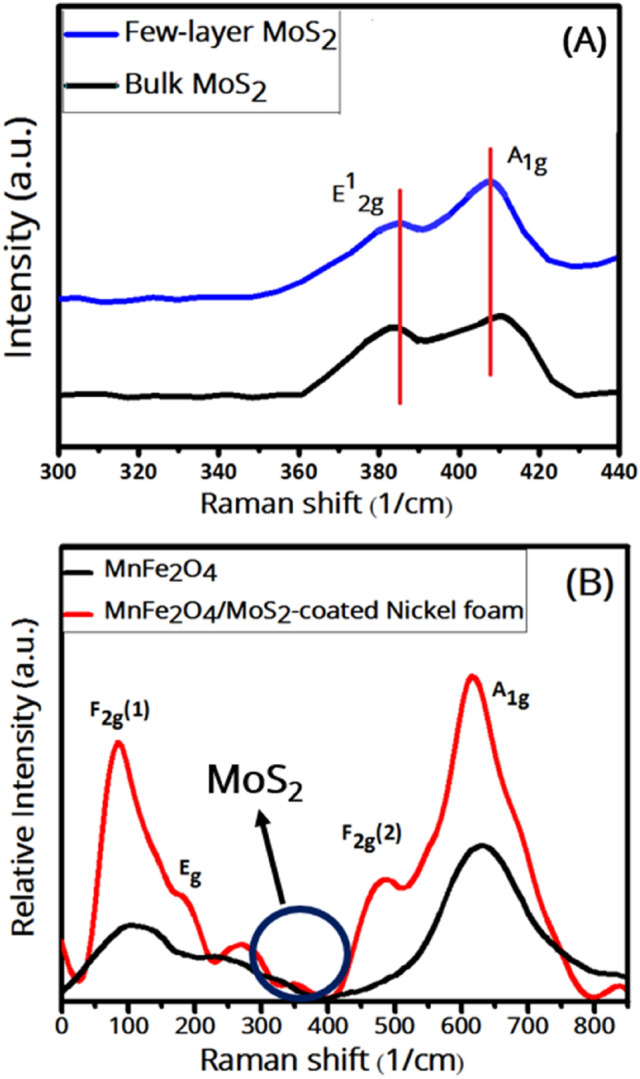
Raman spectra of (**A**) MnFe_2_O_4_ nanoparticles, and MnFe_2_O_4_/MoS_2_-coated Ni foam, (**B**) few-layer MoS_2_ nanosheets.

Figure 3 shows the Raman spectra of the samples. The Raman spectrum of few-layer MoS_2_ nanosheets (Fig. 3A) show two peaks at 385 and 407 cm^-1^ attributed to the in-plane (E_2g_) and the out-of-plane (A_1g_) vibration modes, respectively, of few-layer MoS_2_^32^. In the Raman spectrum of the bulk MoS_2_ powder, the lower-wavenumber mode shifts slightly towards a lower wavenumber (387 cm^−1^), and the higher-wavenumber mode shifts slightly towards a higher wavenumber (411 cm^−1^). The shifts are consistent with layer-dependent Raman modes of MoS_2_ sheets^42^, confirming that our exfoliated MoS_2_ nanosheets are few-layer. In the Raman spectrum of MnFe_2_O_4_ nanoparticles (Fig. 3B), the E_g_ band (189 cm^−1^) is due to the asymmetric and symmetric bending of O with respect to Fe, the F_2g_(1) band (107 cm^−1^) is due to the translational movement of the whole tetrahedron (FeO_4_), and the A_1g_ band (630 cm^−1^) is due to the symmetric stretching of oxygen atoms along Fe–O (or Mn–O) tetrahedral bonds^14^. The bands confirm the inverse spinel structure of the MnFe_2_O_4_^43^. The Raman spectrum of the MnFe_2_O_4_/MoS_2_ nanocomposite coated on a nickel foam shows the peaks due to MnFe_2_O_4_ and also the F_2g_(2) band, which is due to the asymmetric stretching of Fe/Mn–O bonds, as well as the E_2_g band of few-layer MoS_2_ nanosheets, confirming the presence of both MnFe_2_O_4_ and MoS_2_ in the nanocomposite. Here, the peaks due to MnFe_2_O_4_ have shifted toward lower wavenumbers as compared to the pure ferrite nanoparticles (617, 484, 195, and 86 cm^−1^ corresponding to A_1g_, F_2g_(1), E_g_, and F_2g_(2) modes, respectively), which can be due to the applied strain when they are composited with MoS_2_ nanosheets. On the other hand, no Raman mode is expected from the nickel foam, as all metals with one atom per unit cell, like Ni, are Raman inactive^44^.

**Figure 4:**
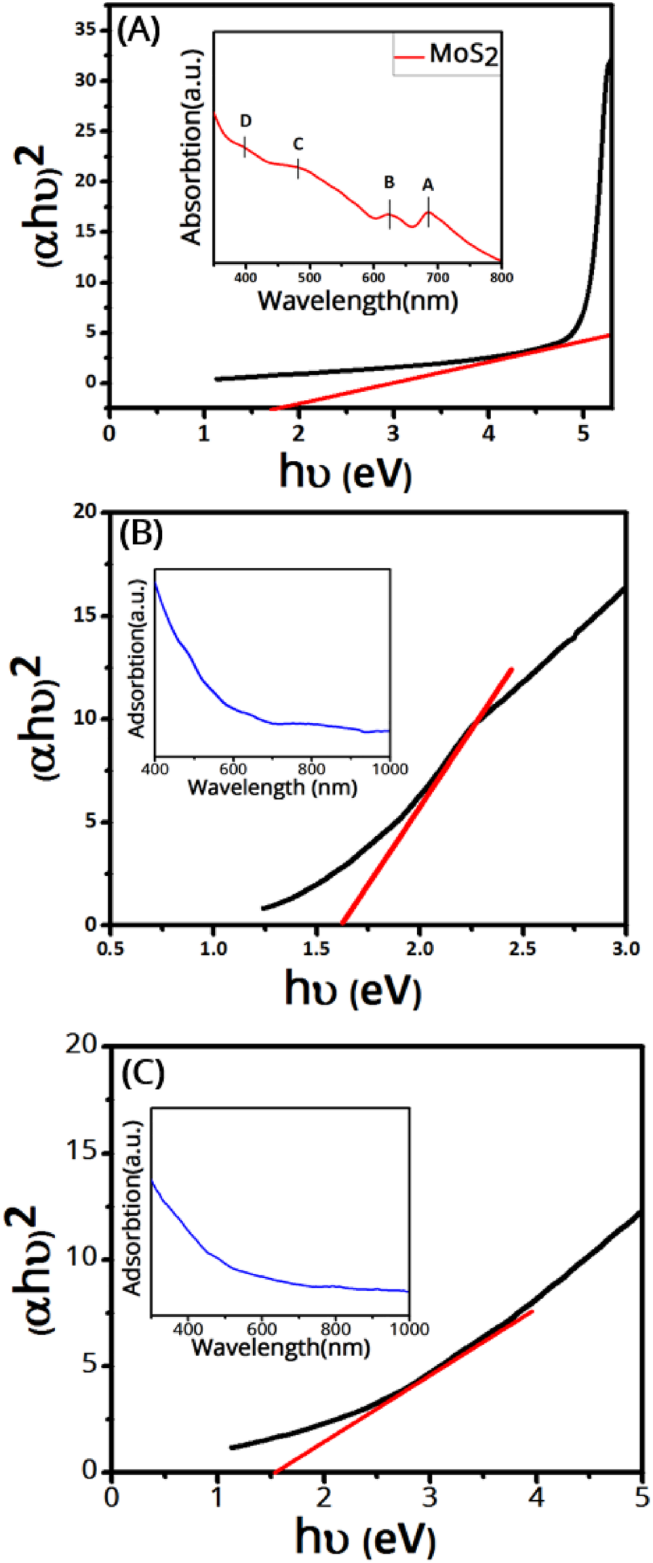
UV–Vis absorption spectra and Tauc plots (insets) of (**A**) MoS_2_ nanosheets, (**B**) MnFe_2_O_4_ nanoparticles, and (**C**) the MnFe_2_O_4_/MoS_2_ nanocomposite.

UV–vis absorption spectroscopy is a powerful tool to investigate the optical properties of semiconductor materials^45^. UV–Vis absorption spectra of the samples with their corresponding Tauc plots are shown in Fig. 4. Optical bandgaps of the samples were estimated using the classical Tauc relation.

3 $${\left( {\alpha h\nu } \right)^n}\, = \,B\left( {h\nu - {E_g}} \right)$$

where α, ν, n, B, h, and E_g_ is the absorption coefficient, the photon frequency, a constant that depends on the bandgap type (1/2 and 2 for direct and indirect band gaps), a constant, the Planck’s constant, and the optical bandgap, respectively. The optical band gap is estimated from an extrapolation of the linear part of (αhν)^2^ versus the photon energy (hν) for direct bandgaps. The UV–Vis spectrum of MoS_2_ nanosheets shows four characteristic peaks at 684, 625, 481, and 399 nm, corresponding to four different electronic transitions denoted with A, B, C, and D, consistent with previously reported values^33^. The few-layer MoS_2_ nanosheets exhibit a bandgap of 1.7 eV, consistent with the literature^33^. The MnFe_2_O_4_ sample shows a bandgap of 1.6 eV^14^. It is observed that the bandgap of the MnFe_2_O_4_/MoS_2_ nanocomposite is almost smaller than that of the pure MnFe_2_O_4_ nanoparticles. This is due to the creation of intermediate states between the valence band and the conduction band of MnFe_2_O_4_ resulting from MoS_2_. The lower bandgap of the MnFe_2_O_4_/MoS_2_ nanocomposite as compared to the pure MnFe_2_O_4_ nanoparticles and the presence of intermediate states can increase the conductivity of the nanocomposite which in turn can enhance its capacitance.

**Figure 5:**
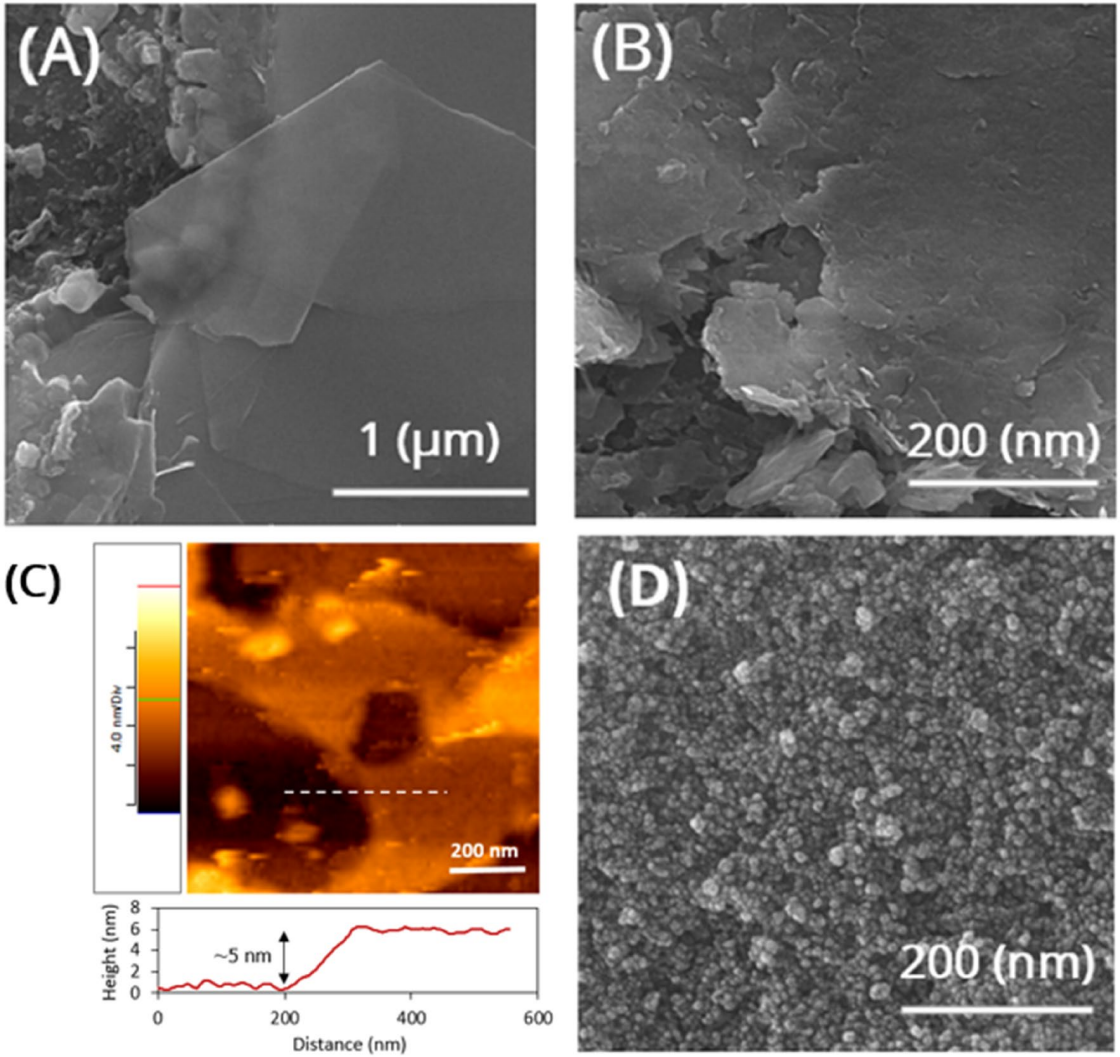
(**A**,**B**) FESEM and (**C**) AFM images of exfoliated few-layer MoS_2_ nanosheets. (**D**) A FESEM image of the MnFe_2_O_4_ nanoparticles.

FESEM images of the prepared samples are shown in Fig. 5. The pure MoS_2_ nanosheets in Fig. 5A,B are sufficiently wide with a lateral size of about 2 to 3 µm, which is appropriate as a substrate to hold other nanoparticles. Some obvious foldings can be seen in these MoS_2_ nanosheets, and one can conclude that they are few-layer. Figure 5C shows an AFM micrograph of an exfoliated MoS_2_ nanosheets on a mica substrate. According to the height profile of the shown dashed line drawn, the thickness of the sheet is ~ 5 nm, indicating that the exfoliated MoS_2_ nanosheets are few-layer. Figure 5D shows that the MnFe_2_O_4_ nanoparticles are highly uniform in size with an average diameter of ~ 10 nm, which is very close to the size calculated from the XRD spectrum (~ 9.5 nm).

**Figure 6:**
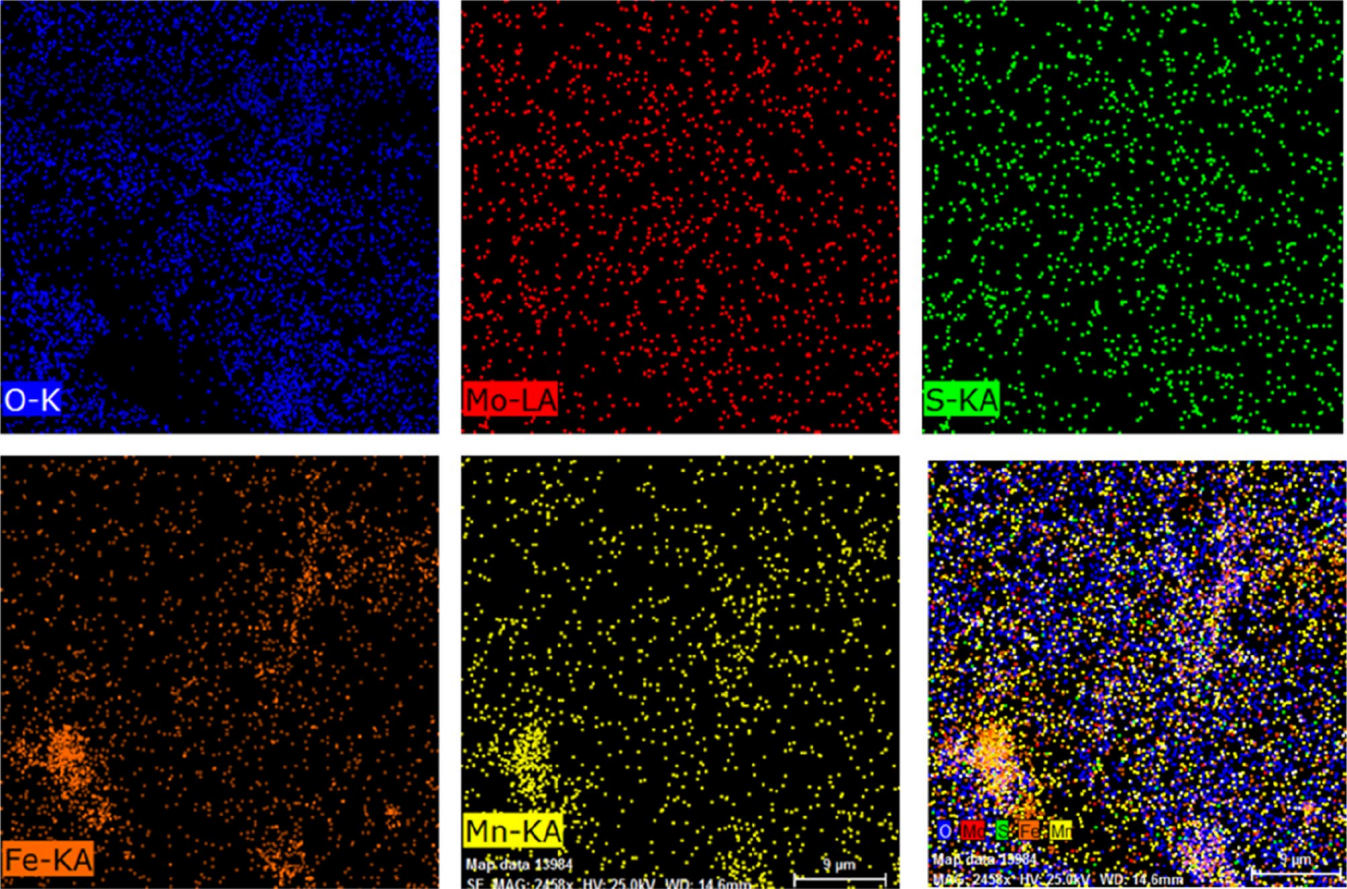
EDS mapping for different atoms in the MnFe_2_O_4_/MoS_2_ nanocomposite.

The elemental composition and distribution of different atoms in the prepared MnFe_2_O_4_/MoS_2_ nanocomposite (MnFe_2_O_4_ nanoparticles attached on MoS_2_ nanosheets) were also analyzed by energy-dispersive X-ray spectroscopy (EDS) mapping, shown in Fig. 6. It is seen that Mo and S atoms are uniformly distributed on the entire mapped window, indicating the presence of MoS_2_ nanosheets. Furthermore, the distribution of Mn, Fe, and O atoms are almost uniform and similar, with some aggregates of them in the lower-left part of the mapped window. This uniform and dense distribution of the ferrite nanoparticles enabled by the presence of MoS_2_ nanosheets can provide a larger surface to volume ratio, required for an enhanced charge transfer and, in turn, a higher capacitance.

**Figure 7:**
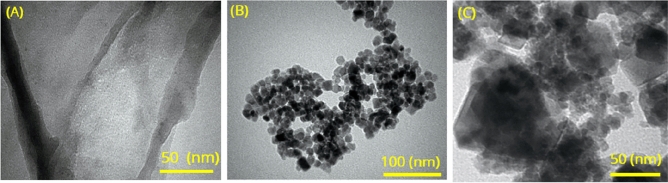
TEM images of (**A**) few-layer MoS_2_ nanosheets, (**B**) MnFe_2_O_4_ nanoparticles, and (**C**) the MnFe_2_O_4_/MoS_2_ nanocomposite.

Figure 7 shows TEM images of the few-layer MoS_2_ nanosheets, the MnFe_2_O_4_ nanoparticles, and the MnFe_2_O_4_/MoS_2_ nanocomposite. Figure 7A shows a few-layer MoS_2_ nanosheet with some wrinkles, indicating its low thickness. The MnFe_2_O_4_ nanoparticles in Fig. 7B are nearly uniform in diameter, with a mean diameter of ~ 13 nm, which is very close to the sizes found from XRD calculations and FESEM measurements. Figure 7C shows the MnFe_2_O_4_/MoS_2_ nanocomposite, where both MoS_2_ nanosheets and MnFe_2_O_4_ nanoparticles can be seen.

**Figure 8:**
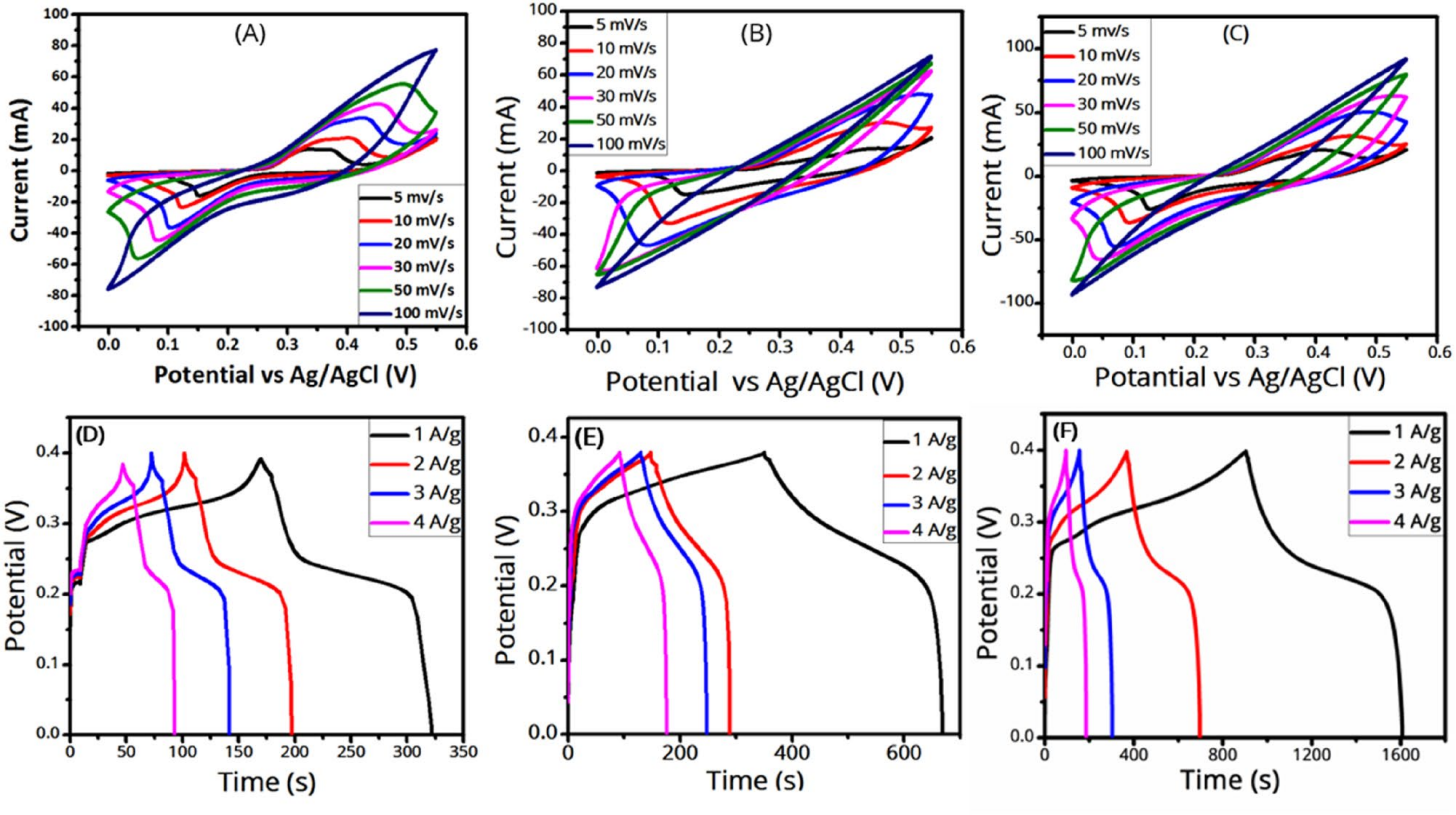
CV (at various scan rates) and GCD (at various current densities) curves of (**A**,**D**) few-layer MoS_2_ nanosheets, (**B**,**E**) MnFe_2_O_4_ nanoparticles, and (**C**,**F**) the MnFe_2_O_4_/MoS_2_ nanocomposite.

Figure 8 shows the CV curves of the samples at various scan rates, where two peaks are seen with positive and negative currents corresponding to oxidation and reduction processes at the electrode’s surface, respectively^46^. As it is seen, by increasing the scan rate the oxidation and reduction peaks shift to higher and lower potentials, respectively, because a shorter time would be available for the electrolyte ions to access the electrode’s surface. However, there is a trade-off between potential and time. Besides, at higher scan rates, both the area under the CV curve and the current increase. Nevertheless, it is seen that the area enclosed in a CV curve (or equivalently, the specific capacitance) decreases as the scan rate increases. This is because at higher scan rates, due to the fast migration of ions, some parts of the active surface areas become inaccessible for the charge storage process^14,18,47^. Figure 8 also represents the galvanostatic charge/discharge (GCD) curves of the samples at different current densities in a potential window of 0 to 0.4 V. It is seen that the discharge time of the samples decreases as the current density increases, explained above. The specific capacitance of the electrodes was calculated from their discharge curves according to the equation^14,18^:

4 $${C}_{sp}=2I\frac{\int V dt}{m {\left(\Delta V\right)}^{2}}$$

at different current densities, where C_sp_, I/m, $\int V dt$, and ΔV are the specific capacitance (F/g), the current density (A/g), the area under the discharge curve, and the active potential window, respectively. It should be noted that for battery-type materials, where they have a plateau during their charging and discharging, the capacity should not be calculated using Eq. (4)^34^. However, in our GCD curves, there is no plateau, and instead, an oblique part is seen, consistent with a mostly-pseudocapacitive behavior. Such behavior is more observable in our two-electrode cell measurements (in Fig. 10). Therefore, it is safe to use the formula to calculate the specific capacitance values. The specific capacitances of the samples are reported in Table 1.

**Table 1:** Specific capacitance values of the samples at various current densities.

Current density (A/g)	Specific capacitance (F/g)		
	MoS_2_ nanosheets	MnFe_2_O_4_	MnFe_2_O_4_/MoS_2_
1	440	600	2093
2	428	520	1982
3	415	453	1870
4	346	375	1810

**Figure 9:**
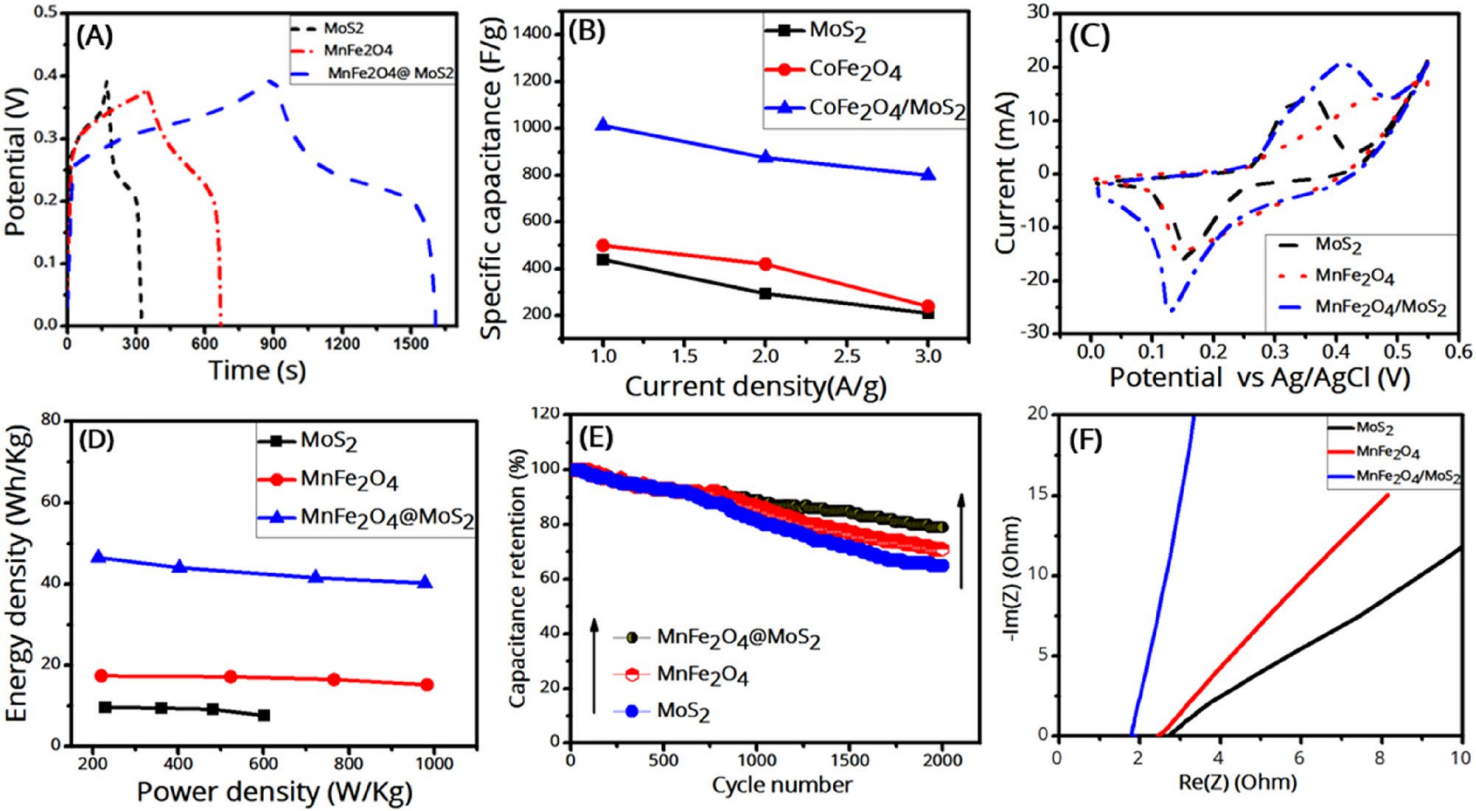
(**A**) GCD curves of the samples at the current density of 1 A/g, (**B**) specific capacitance versus current density for the samples, (**C**) CV curves of the samples at the scan rate of 5 mV/s, (**D**) Ragone plots (energy density versus power density) of the samples, (**E**) cycling stabilities of the samples during 2000 GCD cycles at the current density of 20 A/g, and (**F**) EIS curves of the samples.

As it is seen in Fig. 9A, which compares the GCD curves of the samples at the current density of 1 A/g, the discharge time, or equivalently the specific capacitance, of the MnFe_2_O_4_/MoS_2_ composite is more than those of both MoS_2_ and MnFe_2_O_4_. This can be for several reasons. Firstly, MoS_2_ is itself an active material for electrochemical reactions due to its electronic structure as well as layered structure, because it can accommodate electrolyte ions in its layered structure. Secondly, due to its versatile redox states, MnFe_2_O_4_ has a pseudocapacitive behavior. Therefore, both MoS_2_ and MnFe_2_O_4_ contribute to the supercapacitance of the MnFe_2_O_4_/MoS_2_ nanocomposite. On the other hand, MoS_2_ nanosheets prevent the MnFe_2_O_4_ from agglomeration and aggregation. Similarly, MnFe_2_O_4_ nanoparticles prevent the MoS_2_ nanosheets from restacking. This will increase the active surface area for charge storage processes. In the next section on DFT calculations, we will see how MoS_2_ can redistribute the stored charges on MnFe_2_O_4_ to achieve an improved capacitance. As can be seen in Fig. 9B, the specific capacitance decreases as the current density increases, which is due to the ion diffusion mechanism. In other words, at a lower current density, the electrolyte ions have enough time to penetrate into the active sites on the electrode material, leading to a higher specific capacitance^14^. Figure 9C compares the CV curves of the prepared electrodes at the scan rate of 5 mV/s. The reduction and oxidation peaks are seen around 0.14 V and 0.4 V, respectively. As it is seen, the MnFe_2_O_4_/MoS_2_ nanocomposite shows a larger CV-enclosed area than that of MnFe_2_O_4_, which is itself larger than that of MoS_2_. This could indicate the higher capacitance of the MnFe_2_O_4_/MoS_2_ nanocomposite than MnFe_2_O_4_. The incorporation of MoS_2_ nanosheets largely increased the specific capacitance of MnFe_2_O_4_ from 600 to 2093 F/g at 1 A/g. The power densities of the MoS_2_, MnFe_2_O_4_, and MnFe_2_O_4_/MoS_2_ samples were obtained at the current density of 1 A/g, listed in Table 2, and their Ragone plots (energy density versus power density) are shown in Fig. 9D. The energy and power densities of the samples were calculated based on

5 $$E=\frac{1}{2} {C}_{sp} {\Delta V}^{2}$$

6 $$P=\frac{E}{t}$$

respectively, where ΔV and t are the potential windows and the discharge time (h)^48^. Cyclic stability tests were performed at the current density of 20 A/g for 2000 GCD cycles, as shown in Fig. 9E. It is seen that the incorporation of MoS_2_ nanosheets considerably improved the cycling stability of the pure MnFe_2_O_4_ nanoparticles. This can be due to the fact that MoS_2_ prevents the MnFe_2_O_4_ nanoparticles from detaching from the electrode into the electrolyte, which can improve the capacitance stability of the composite. Electrochemical impedance spectroscopy (EIS) was used to analyze the resistance information of samples. The frequency range of the impedance measurements is 10 MHz–100 kHz. An EIS curve typically consists of two parts: (1) the high-frequency region is a semicircle and (2) the low-frequency region is a straight line, indicating the charge-transfer resistance, and an inclined line, indicating the diffusion of ions into the electrolyte^49,50^. According to the EIS plots in Fig. 9F, the charge transfer resistance of the samples is negligible. The internal resistance can be obtained from the slope of the curves intersecting the x-axis. According to the EIS plots, the internal resistance of the MnFe_2_O_4_/MoS_2_ nanocomposite is lower than those of MoS_2_ and MnFe_2_O_4_ (also see the DFT section), so it has a higher conductivity suitable for supercapacitance. To sum up, the comparative results show that the MnFe_2_O_4_/MoS_2_ nanocomposite exhibits better capacitive performance compared to both MoS_2_ and MnFe_2_O_4_.

**Table 2:** The calculated energy densities and power densities of the samples at the current density of 1 A/g.

Sample	Power density (W/Kg)	Energy density (Wh/Kg)
MoS_2_	229.73	9.77
MnFe_2_O_4_	224.23	17.43
MnFe_2_O_4_/MoS_2_	213.64	46.51

**Figure 10:**
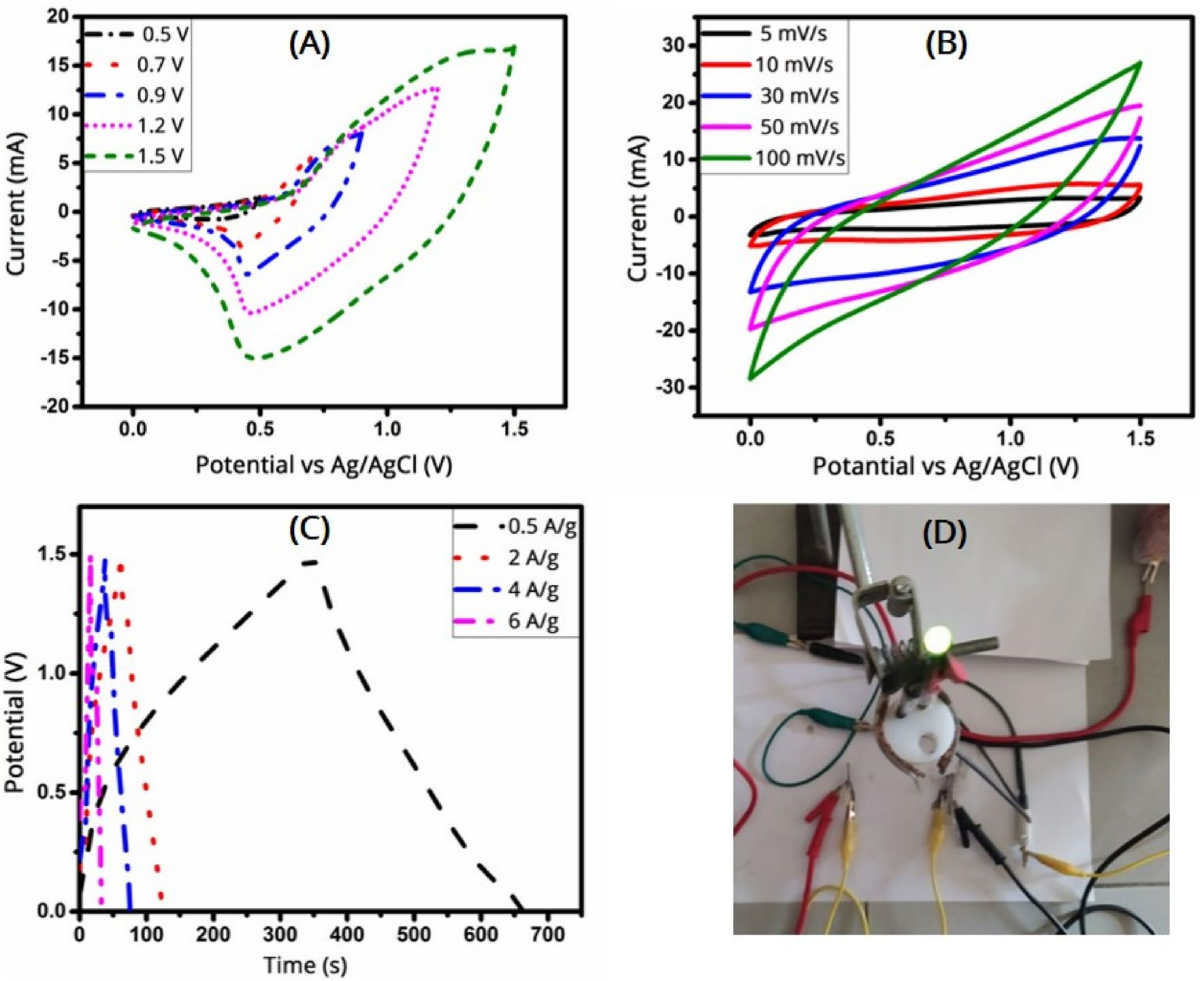
CV curves of the (MnFe_2_O_4_/MoS_2_)//AC asymmetric supercapacitor (**A**) for different potential windows at 20 mV/s and (**B**) for different scan rates at the potential window of 1.5 V. (**C**) GCD curves of the supercapacitor at different current densities at the potential window of 1.5 V. (**D**) A picture of the assembled asymmetric supercapacitor lighting up a green LED.

Next, we assembled the (MnFe_2_O_4_/MoS_2_)//AC asymmetric supercapacitor in a two-electrode setup, as discussed in the “Experimental” section. Figure 10A shows the CV curves of the device for incremental voltages to confirm its operating potential. It is seen that the capacity increases as the potential window increases, indicating the ability of the device to perform faradaic processes at higher voltages. Figure 10B shows the CV curves of the device for various scan rates at the potential window of 0–1.5 V, showing quasi-rectangular shapes that illustrate the good electrochemical reversibility of the device. Figure 10C shows the GCD curves of the device for various current densities. The charge and discharge parts of the GCD curves are almost symmetric, which demonstrates a small internal resistance drop, indicating the contributions from both the faradaic processes and the double layer capacitance^51^. Figure 10D illustrates the lighting up of a green light-emitting diode (LED) using the (MnFe_2_O_4_/MoS_2_)//AC asymmetric supercapacitor.

## DFT study

**Figure 11:**
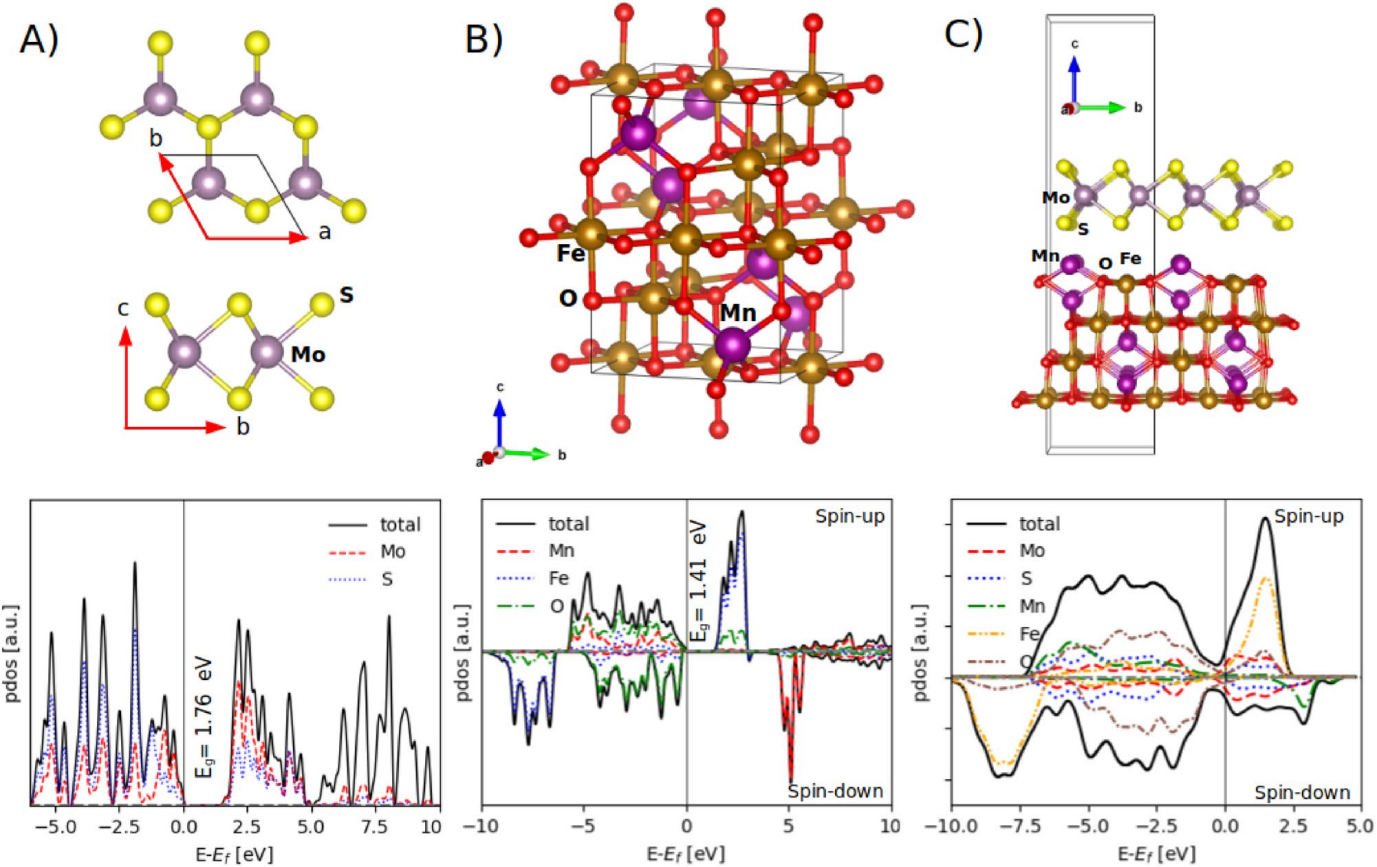
The unit cells and the atom-projected density of states of (**A**) the MoS_2_ monolayer, (**B**) the normal spinel MnFe_2_O_4_, and (**C**) the MnFe_2_O_4_/MoS_2_ interface. The crystal structure images were produced by VESTA (version 3.4.5)^39^.

MoS_2_ is a layered transition metal dichalcogenide where its layers are bound together by weak van der Waals (vdW) forces. Each MoS_2_ monolayer consists of three atomic layers in the sequence of S-Mo-S. Bulk MoS_2_ is a p-type indirect-bandgap semiconductor (1.23 eV), and its bandgap slightly increases to 1.8 eV as the number of layers decreases to one^52,53^. Figure 11A shows top and side views of the optimized structure of the MoS_2_ monolayer and its atom-projected density of states. We found the optimized lattice constant of 3.18 Å and the Mo-S bond length of 2.41 Å, and the MoS_2_ monolayer shows a direct bandgap of 1.76 eV at the K point, consistent with^53^. On the other hand, MnFe_2_O_4_ is an insulating, soft ferrimagnetic spinel ferrite, which crystallizes in a mixed-phase spinel structure^54^ with an almost low inversion degree of 0.2, where 80% and 20% of Mn^2+^ ions occupy the tetrahedral sites and octahedral sites, respectively, and Mn^2+^ and Fe^3+^ ions are distributed in the remaining tetrahedral and octahedral sites. Recently, we compared the experimental and theoretical results of MnFe_2_O_4_ and showed that the true XRD pattern of MnFe_2_O_4_ is a combination of normal and inverse spinel XRD patterns^14^. However, for the sake of simplicity, we considered here the normal spinel configuration for MnFe_2_O_4_ (see Fig. 11B). We considered a 28-atom unit cell for the bulk MnFe_2_O_4_ as half of a simple cubic structure. Figure 11B shows the unit cell and the atom-projected density of states of the bulk MnFe_2_O_4_. It is seen that the structure is an insulator with a direct bandgap of 1.41 eV. The *a* and *c* lattice constants were found 6.14 and 8.68 Å, respectively.

Next, we created the MnFe_2_O_4_/MoS_2_ interface (see Fig. 11C). The lattice mismatch between the MnFe_2_O_4_ surface and the MoS_2_ monolayer was ~ 5%. We applied the strain to the MoS_2_ monolayer because it only affects its bandgap and cannot change its semiconducting nature^55^. In the optimized structure, the smallest distance between Mn and S atoms is 2.79 Å, which is larger than the sum of the covalent radii of both atoms (1.39 and 1.02 Å for Mn and S, respectively), indicating that the coupling between the MnFe_2_O_4_ slab and the MoS_2_ monolayer is of the vdW type, not covalent. The binding energy between the MoS_2_ monolayer and the MnFe_2_O_4_ slab is defined as:

7 $${E}_{b}={E}_{interface}-{E}_{Mn{Fe}_{2}{O}_{4}}-{E}_{{MoS}_{2}}$$

where E_b_ is the binding energy and ${E}_{Mn{Fe}_{2}{O}_{4}}$, ${E}_{{MoS}_{2}}$, and ${E}_{interface}$ are the total energies of the MnFe_2_O_4_/MoS_2_ interface, the MoS_2_ monolayer, and the MnFe_2_O_4_ slab, respectively. The binding energy (E_b_) was calculated -1.44 eV per unit cell, indicating the physisorption nature of the coupling between the MnFe_2_O_4_ slab and the MoS_2_ monolayer. The interlayer distance of 3.19 Å confirms again the vdW nature of the coupling between the individual layers. This indicates that the MoS_2_ monolayer would not have any significant influence on the electronic properties of MnFe_2_O_4_ and just creates some energy levels near its Fermi level and redistributes the charge density of MnFe_2_O_4_ at its surface.

**Figure 12:**
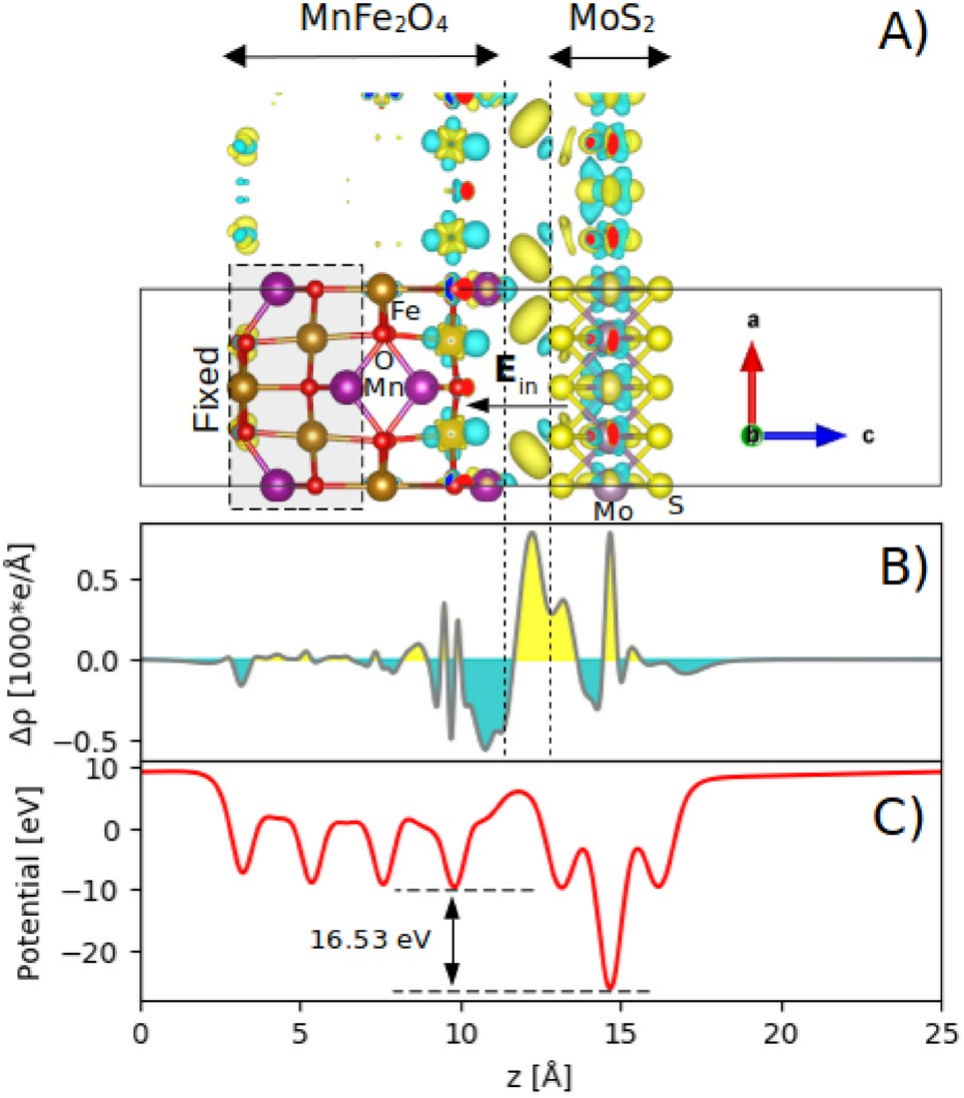
(Color online) (**A**) A 3D picture of the differential charge density of the MnFe_2_O_4_/MoS_2_ interface, where the yellow and blue areas denote the gain and the loss of electrons, respectively, with the isosurface value of 0.016 e/Å^3^, with (**B**) its corresponding in-plane averaged differential charge density along the z-direction. The space between the dashed lines contains no atom. (**C**) The in-plane averaged electrostatic potential of the MnFe_2_O_4_/MoS_2_ interface.

Figure 12A shows the differential charge density of the MnFe_2_O_4_/MoS_2_ interface (i.e., the charge density of the MnFe_2_O_4_/MoS_2_ interface minus those of the isolated MnFe_2_O_4_ slab and the isolated MoS_2_ monolayer). It is seen that the electrons just below the MnFe_2_O_4_ surface have been depleted, while they have been accumulated on the MoS_2_ surface, more on the nearest sulfur layer, which is due to the higher electron affinity of S as compared to Mo. The integrated charge density difference was calculated using the following equation:

8 $$\Delta \rho \left(z\right)=\int {\rho }_{interface}dxdy-\int {\rho }_{Mn{Fe}_{2}{O}_{4}}dxdy-\int {\rho }_{{MoS}_{2}}dxdy$$

where ${\rho }_{interface}$, ${\rho }_{Mn{Fe}_{2}{O}_{4}}$, and ${\rho }_{{MoS}_{2}}$ denote the charge densities corresponding to the MnFe_2_O_4_/MoS_2_ interface, the MnFe_2_O_4_ slab, and the MoS_2_ monolayer. The result is shown in Fig. 12B. The net charge transfer from the MnFe_2_O_4_ slab to the MoS_2_ monolayer was calculated as 2 electrons. Figure 12C shows the in-plane averaged electrostatic potential of the MnFe_2_O_4_/MoS_2_ interface along the z-direction. It is seen that the interface develops a very large difference (16.53 eV) in the in-plane averaged electrostatic potential across the interface, leading to a large intrinsic built-in electric field (E_in_) from MoS_2_ to MnFe_2_O_4_. This large built-in electric field drives electrons from MnFe_2_O_4_ towards MoS_2_, enhancing the interlayer coupling. The attraction of charge density from MnFe_2_O_4_ to MoS_2_ would help to enhance the charge storage of the composite. When charging, due to the constant transfer of electrons from the electrolyte to MnFe_2_O_4_ and in turn to MoS_2_, a longer time would be needed to reach the charge saturation state (as seen in GCD curves in Fig. 9). When discharging, the process is reversed and a long time would be needed for a fully discharged state (again consistent with GCD curves in Fig. 9). This can enhance the specific capacitance of the pure MnFe_2_O_4_.

To sum up, according to the hybrid experimental and computational work, one can conclude that MoS_2_ can enhance the charge storage capability and the specific capacitance of MnFe_2_O_4_ for several reasons: (1) MoS_2_ itself can exhibit a supercapacitance behavior, (2) MoS_2_ nanosheets will act as a substrate to hold MnFe_2_O_4_ nanoparticles uniformly so that they will not be agglomerated, (3) in a similar manner, the MnFe_2_O_4_ nanoparticles can prevent the MoS_2_ nanosheets from restacking, (4) MoS_2_ nanosheets provide a significantly higher active surface area, (5) MoS_2_ would create several energy levels near the Fermi energy of MnFe_2_O_4_ that are suitable for charge storage, and (6) MoS_2_ will attract the electron charge density from MnFe_2_O_4_ and constantly redistribute the stored charges. These all can synergistically enhance the specific capacitance of pure MnFe_2_O_4_ nanoparticles.

## Conclusions

MnFe_2_O_4_ nanoparticles were in-situ synthesized on pre-exfoliated few-layer MoS_2_ nanosheets via a simple hydrothermal method, and the synthesized MnFe_2_O_4_/MoS_2_ nanocomposite was studied for supercapacitor applications. We found that owing to the effect of MoS_2_, the MnFe_2_O_4_/MoS_2_ nanocomposite demonstrates a considerably higher (~ 3.5 times) specific capacitance and better charge–discharge cycling stability as compared to pure MnFe_2_O_4_. Using DFT calculations, we attributed the improvement to the energy levels of MoS_2_ near the Fermi level of the composite, making it a conductor, and the attraction of electron charge density from MnFe_2_O_4_ to MoS_2_, which will help the redistribution of electrons between MoS_2_ and MnFe_2_O_4_ when charging and discharging. Figure S1 (in the supplementary material) provides a summary schematic of the research findings.

## Supplementary Information

Supplementary Information

## Data Availability

The datasets generated during and/or analyzed during the current study are available from the corresponding author on reasonable requests.
