# Supplementary material for: Highly improved supercapacitance properties of MnFe2O4 nanoparticles by MoS2 nanosheets
Source: Sci Rep. 2021 Apr 16;11:8378. doi: 10.1038/s41598-021-87823-6 (PMC8052405; doi:10.1038/s41598-021-87823-6)
Supplement: Supplementary file 1 — Supplementary Information [file 41598_2021_87823_MOESM1_ESM.docx]

**Highly improved supercapacitance properties of MnFe_2_O_4_ nanoparticles by MoS_2_ nanosheets**

**Samira Sharifi, Kourosh Rahimi, and Ahmad Yazdani***

Condensed Matter Physics Group, Department of Basic Sciences, Tarbiat Modares University, Jalal-Ale-Ahmad Avenue, Tehran, Iran.

*Corresponding author: yazdania@modares.ac.ir

Figure S1 presents a graphical abstract of the research highlights.


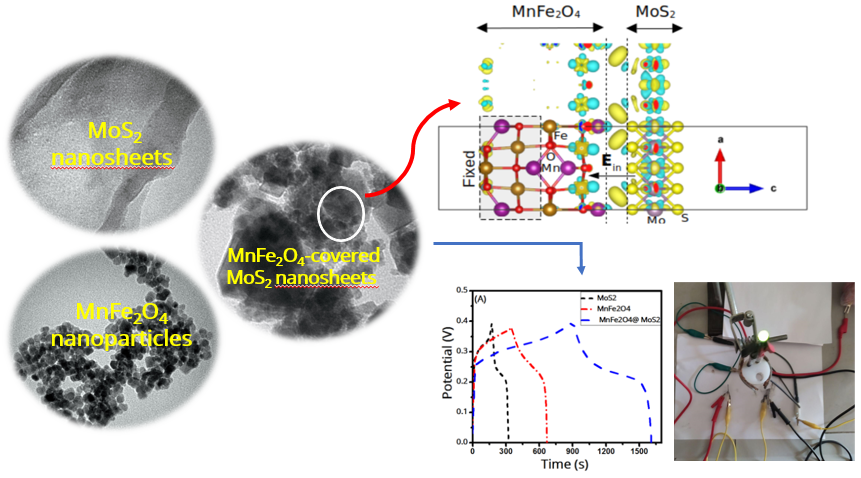


**Figure S1.** Graphical abstract of the research
